# Ambulatory care for HIV-infected patients: differences in outcomes between hospital-based units and private practices: analysis of the RESINA cohort

**DOI:** 10.1186/2047-783X-18-48

**Published:** 2013-11-21

**Authors:** Mark Oette, Stefan Reuter, Rolf Kaiser, Björn Jensen, Thomas Lengauer, Gerd Fätkenheuer, Heribert Knechten, Martin Hower, Abdurrahman Sagir, Herbert Pfister, Dieter Häussinger

**Affiliations:** 1 Clinic for General Medicine, Gastroenterology, and Infectious Diseases Augustinerinnen Hospital, Jakobstrasse 27-31, Koln 50678, Germany; 2 Klinikum Leverkusen, Leverkusen, Germany; 3 University of Cologne, Cologne, Germany; 4 University Clinic Düsseldorf, Düsseldorf, Germany; 5 Max Planck Institute for Informatics, Saarbrücken, Germany; 6 Private Practice Blondelstrasse, Aachen, Germany; 7 Klinikum Dortmund, Dortmund, Germany

**Keywords:** HAART, Therapy naïve, Transmitted drug resistance, Treatment setting

## Abstract

**Background:**

The efficacy of highly active antiretroviral therapy (HAART) in the treatment of HIV infection is influenced by factors such as potency of applied drugs, adherence of the patient, and resistance-associated mutations. Up to now, there is insufficient data on the impact of the therapeutic setting.

**Methods:**

Since 2001, the prospective multicenter RESINA study has examined the epidemiology of transmitted HIV drug resistance in Nordrhein-Westfalen, the largest federal state of Germany by population. Characteristics of patients treated in hospital-based outpatient units were compared to those of patients treated in medical practices. Longitudinal data of all participants are being followed in a cohort study.

**Results:**

Overall, 1,591 patients were enrolled between 2001 and 2009 with follow-up until the end of 2010. Of these, 1,099 cases were treated in hospital-based units and 492 in private practices. Significant differences were found with respect to baseline characteristics. A higher rate of patients with advanced disease and non-European nationality were cared for in hospital units. Patients in medical practices were predominantly Caucasian men who have sex with men (MSM) harboring HIV-1 subtype B, with lower CDC stage and higher CD4 cell count. Median viral load was 68,828 c/mL in hospital-based units and 100,000 c/mL in private practices (*P* = 0.041). Only median age and rate of transmitted drug resistance were not significantly different. After 48 weeks, 81.9% of patients in hospital units and 85.9% in private practices had a viral load below the limit of detection (*P* = 0.12). A similar result was seen after 96 weeks (*P* = 0.54). Although the baseline CD4 cell count was different (189.5/μL in hospital units and 246.5/μL in private practices, *P* <0.001), a consistent and almost identical increase was determined in both groups.

**Conclusions:**

The RESINA study covers a large HIV-infected patient cohort cared for in specialized facilities in Germany. Despite significant differences of patients’ baseline characteristics in hospital-based units compared to medical practices, we could not find significant differences in treatment outcome up to 2 years after the initiation of HAART.

## Background

Currently, there are approximately 78,000 HIV-infected persons in Germany, and about 3,000 individuals are newly infected each year. More than 50,000 patients receive highly active antiretroviral therapy (HAART) [1]. After more than a decade of widespread application of HAART, the life expectancy of HIV-infected patients has approached the level of the non-infected population [2]. In general, efficacy of first-line HAART exceeds 80%, measured by the proportion of patients with a viral load below the detection limit [3]. But even after repeated treatment modifications including a variety of substance combinations, the rate of successful therapies has improved continuously over recent years [4]. Therefore, a viral load below the detection limit is currently one of the cornerstones of patient management. As long as eradication of HIV infection is not achievable, HAART still has to be applied continuously for the lifetime of the patient.

The essential determinants of sustained inhibition of viral replication are: potency of applied drugs, immunological host factors, resistance of the virus, and patient aspects such as feasibility of the drug combination as well as adherence [5]. Little is known about the association of medical care setting for treatment success. There is, for example, data on cost-effectiveness of HIV care in private practices versus public hospitals in South Africa [6]. Another study from Switzerland focused on the improvement of co-operation between different facility types [7]. To our knowledge, there are, to date, no outcome results relevant for medical prognosis of patients considering medical care setting.

In this study, we present data on epidemiological characteristics and treatment outcome of HIV-infected patients considering medical facility type in Germany. The structure of HIV care in Germany is diverse. In the ambulatory setting, numerous medical practices as well as hospital-based units offer care for HIV-infected patients, with free access to each type according to patients’ wishes. Quality assurance regulations ensure a high standard of medical management.

The present analysis was extracted from the RESINA study, a prospective cohort of HIV-infected patients after initiation of HAART.

## Methods

The RESINA study is an ongoing prospective multicenter investigation on the epidemiology of transmitted drug resistance in chronically HIV-infected patients in Nordrhein-Westfalen, the largest federal state of Germany by population [8]. Since 2001, more than 2,500 patients were recruited in 36 centers (see Appendix). Follow-up after initiation of first-line HAART is being performed in a longitudinal cohort. All centers are specialized in HIV care. The dataset served for the development of treatment outcome prediction tools using bioinformatic analyses of complex mutational patterns [9]. The RESINA study is part of the nationwide project ‘Monitoring of resistant HIV in newly infected and chronically infected patients in Germany’, with collaboration from the Robert Koch Institute, the Paul-Ehrlich-Institute, and the RESINA study group. Further co-operation with European multicenter projects such as the EuResist Network and the Collaborative HIV and Anti-HIV Drug Resistance Network (CHAIN) are being implemented.

The present analysis covers patients who were included in the study between 2001 and 2009, with available follow-up data to at least 48 weeks and end of 2010. Inclusion criteria of the study were: documented HIV-1 infection and the decision of physician and patient on the initiation of first-line HAART, indicated by virological, immunological, or clinical aspects. Exclusion criteria were: previous application of antiretrovirals and non-willingness to participate. All relevant institutional review boards of cooperating centers rendered positive approval.

Genotypic resistance testing and interpretation of results was applied using geno2pheno (Max Planck Institute for Informatics, Saarbrücken, Germany) as previously described [10]. For test interpretation, the current World Health Organization (WHO) mutation list for the classification of primary or transmitted drug resistance was additionally used to provide clinically relevant advice [11]. Each doctor had a test result available for the treatment choice.

The whole study population was divided into patients treated in outpatient hospital units and patients treated in medical practices. Recruitment into the RESINA study was relevant for inclusion; subsequent movements of the patients between the institutions were not recorded. The following baseline parameters were determined: gender, age, nationality, ethnic origin, HIV transmission group, time since determination of positive HIV test, clinical state using CDC stage, CD4 cell count, HIV load, HIV-1 subtype, and primary drug resistance. The applied regimens were divided into treatment combination types. Treatment outcome was measured by the rate of patients with a viral load below the limit of detection (50 copies/mL) and median amount of CD4 cell count at the time points of 24, 48, 72, and 96 weeks.

For the statistical analysis SPSS version 19 (IBM, Armonk, NY, USA) was used. Univariate comparisons were performed with Wilcoxon rank-sum test or Fisher’s exact test, as appropriate. A p-value of ≤0.05 was regarded as significant; adjustment for multiple testing was not applied. Covariate influence on therapy success as demonstrated by the risk of viral failure after 48 weeks was examined by multivariate analysis using logistical regression. A model considering items with significant distribution differences of baseline characteristics or known impact on treatment outcome was fitted to the data. The parameters of treatment facility, baseline CDC stage and HIV load, transmitted drug resistance, and HIV-1 subtype were included.

## Results

The treatment centers cooperating in the study consisted of four outpatient units of university clinics, four outpatient units of communal hospitals, and 28 medical practices. Altogether, 1,591 patients with available follow-up data up to 48 weeks were recruited in the period from 2001 to 2009. Of these, 1,099 were treated in hospital-based units and 492 in private practices. The baseline characteristics of the population are demonstrated in Table 1. Significant differences in the distribution among the treatment settings were found for gender, nationality, ethnic origin, HIV transmission group, duration of HIV-positivity, CDC stage, CD4 cell count, viral load, and HIV-1 subtype.

**Table 1:** Baseline characteristics of the cohort

**Parameter**	**Total**	**Private practice**	**Hospital-based units**	***P *** **value**
Population (n)	1,591	492	1,099	-
Gender (%)				<0.001
Male	80.0	87.0	76.9	
Female	20.0	13.0	23.1	
Age (years) (median, range)	38 (18 to 78)	38 (19 to 69)	38 (18 to 78)	0.1
Nationality (%)				<0.001 (German versus others)
German	71.9	81.3	67.7	
Thai	2.4	0.8	3.1	
Turkish	2.3	2.0	2.5	
Cameroonian	2.2	0.4	3.0	
Kenyan	2.2	1.8	2.4	
Other (<2%)	18.6	13.3	20.9	
Missing data	0.4	0.4	0.4	
Ethnic origin (%)				<0.001 (Caucasian versus others)
Caucasian	81.2	89.0	77.7	
African	13.3	7.7	15.7	
Asian	3.5	1.0	4.6	
Other (<1%)	1.6	1.7	1.7	
Missing data	0.4	0.6	0.3	
HIV transmission groups (%)				<0.001 (MSM versus others)
MSM	52.5	68.3	45.5	
Heterosexual	18.7	12.2	21.7	
Endemic region	14.6	7.3	17.9	
Intravenous drug abuse	5.7	5.3	5.9	
Others (<1%)	1.6	0.4	2.0	
Missing data	6.9	6.5	7.0	
Duration of HIV-positivity (months) (median, range)	1 (0 to 260)	5 (0 to 260)	1 (0 to 244)	<0.001
CDC stage (%)				0.042 (AIDS versus non-AIDS)
A	39.7	46.0	37.2	
B	26.9	26.5	27.1	
C	33.4	27.5	35.7	
CD4 cell count (CD4 cells/μL) (median, range)	210 (0 to 1,224)	246.5 (0 to 1,224)	189.5 (1 to 1,014)	<0.001
Viral load (copies/mL) (median, range)	75,345.5 (227 to 5,022,690)	100,000 (227 to 5,022,690)	68,828 (508 to 500,000)	0.041
HIV-1 subtype: B	71.9	81.3	67.7	<0.001 (B versus others)
Transmitted drug resistance: resistance demonstrated	9.7	10.8	9.2	0.36 (resistance versus wild type)

Values presented as percentages, unless otherwise indicated. CDC, Centers for Disease Control and Prevention; *MSM*, men who have sex with men.

Overall, 90.3% of patients had standard combinations as defined by European or German-Austrian guidelines. Of these, 49.0% of patients had a combination with two nucleoside reverse transcriptase inhibitors (NRTIs) and a ritonavir-boosted protease inhibitor (PI); 37.4% received a combination of two NRTIs with a non-nucleoside reverse transcriptase inhibitor. For 3.9% of patients, a single PI was combined with two NRTIs. A subset of patients had other combinations (for example more than three active drugs in the regimen (3.6%) in special situations such as transmitted drug resistance).

**Figure 1:**
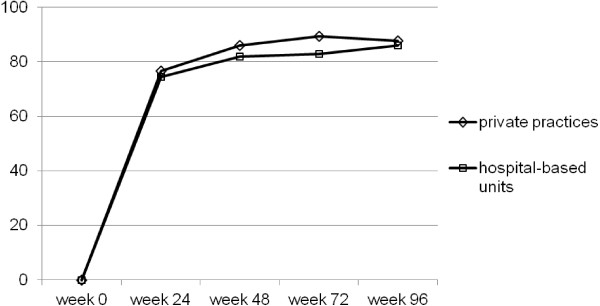
**Virological outcome of therapy.** Rate of patients with a viral load <50 copies/mL.

**Figure 2:**
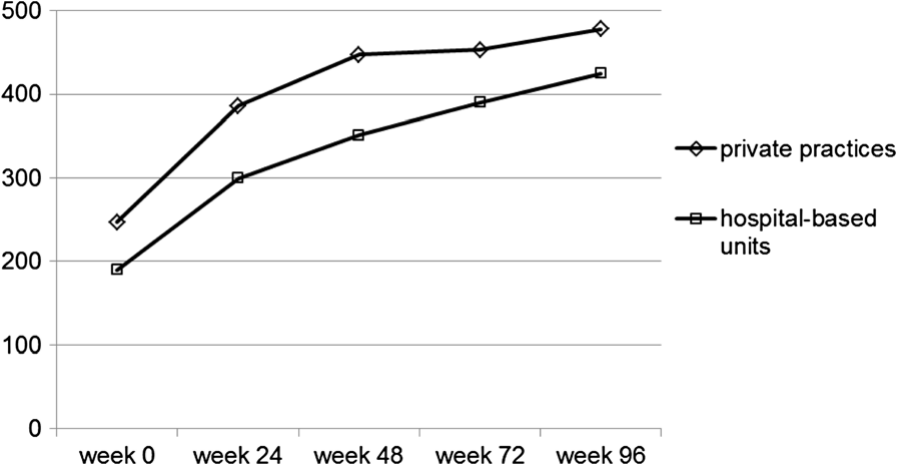
**Immunological outcome of therapy.** Median CD4 cell count (CD4 cells/μL).

Figure 1 shows the rate of patients with effective therapy, as measured by a viral load below the level of detection. For 81.9% (hospital-based units) and 85.9% (medical practices) of patients with a suppressed viral load after 48 weeks there was no significant difference between the different treatment settings (*p* = 0.12). Corresponding values at 96 weeks were 86.0% versus 87.6% (p = 0.54). The development of the CD4 cell count is demonstrated in Figure 2. Despite the different values at baseline and at the follow-up time points (*p* <0.001 for all comparisons), the two graphs show parallel development with a median increase of 235.5 CD4 cells/μL in hospital-based units and 231.5 CD4 cells/μL in private practices (p = 0.62), respectively.

The result of the multivariate analysis is shown in Table 2. Considering the rate of patients with detectable viral load after 48 weeks, only the HIV-specific baseline parameter CDC stage (AIDS versus non-AIDS) was found to have significant impact on therapy efficacy after adjustment of the items: treatment setting, viral load, transmitted drug resistance, and HIV-1 subtype.

**Table 2:** Multivariate analysis considering treatment efficacy

**Parameter**	**OR**	**95% CI**	***P *** **value**
Treatment setting: hospital versus private practice	0.94	0.53 to 1.68	0.85
CDC stage: AIDS versus non-AIDS	**1.82**	**1.09 to 3.06**	**0.023**
HIV load: for each log c/mL	1.2	0.82 to 1.75	0.34
Transmitted drug resistance: resistance demonstrated versus wild type virus	0.81	0.33 to 2.0	0.65
HIV-1 subtype: B versus others	0.72	0.42 to 1.25	0.55

Risk of viral failure (viral load >50 copies/mL) after 48 weeks. CDC, Centers for Disease Control and Prevention; CI, confidence interval; OR, adjusted odds ratio.

## Discussion

The RESINA study is a prospective multicenter trial of HIV therapy in Nordrhein-Westfalen, Germany, which has been running since 2001 [8]. Due to the similarity of the study cohort and the HIV-infected population in Germany [10,12], the inclusion criteria of intended initiation of first-line HAART and the recruitment of more than 1,500 patients, the data may represent current treatment reality of HIV-positive patients. The aim of the investigation was to analyze epidemiological features and treatment outcome results of first-line HAART with regard to patients’ medical care setting. As discussed above, there is no evidence regarding this topic existing to date.

In this study, we found significant differences between the baseline parameters of HIV-infected patients cared for in hospital-based outpatient units and private practices. Patients in hospital-associated settings showed a higher proportion of CDC stage of AIDS, a lower CD4 cell count, and a higher frequency of non-B HIV-1 subtype. Patients who presented to medical practices of general practitioners or infectious disease specialists were predominantly Caucasian men who have sex with men (MSM) of German nationality. Thus, in Germany, the HIV population in ambulatory care is distributed heterogeneously regarding medical facility types. Although the median viral load differed significantly in both groups, we consider the amount of approximately 30,000 copies/mL to be clinically not relevant. This is confirmed by the multivariate analysis, which shows that baseline viral load had no significant impact on therapy outcome. However, the characteristics suggest unequal chances of efficient antiretroviral therapy due to the inferior clinical stage of patients in hospital-based units.

Despite different distribution of baseline predictors of successful HAART, treatment efficacy was comparable in the two groups. The rate of study participants with a viral load below the level of detection was very similar in follow-up, with an overall excellent success rate of well above 80% after 48 weeks and 85% after 96 weeks in the two strata, and no significant difference at both time points. The CD4 cell count was significantly different at baseline, but the increase after initiation of HAART was almost identical in both groups up to 96 weeks. At the end of follow-up, the median CD4 cell count was well above 400/μL in the two populations. Further evaluation of the data using multivariate analysis could not show an association of treatment setting with virological outcome at 48 weeks after adjustment for baseline viral load, transmitted drug resistance, and HIV-1 subtype. Only baseline CDC stage (AIDS versus non-AIDS) was associated with treatment success, a fact that is well-known from clinical practice. As previously demonstrated [13], transmitted drug resistance was not associated with inferior outcome. This is due to the fact that the cases presenting with viral mutations received a combination therapy optimized by resistance test results. In summary, overall virological and immunological efficacy of first-line HAART was very good, independent of medical care facility.

The higher rate of individuals of non-Caucasian ethnic origin and non-European nationality treated in hospital-based units was accompanied by a higher rate of the heterosexual transmission group and a lower rate of non-B HIV-1 subtype. However, the lower proportion of these patients in private practices compared to German patients with a predominantly MSM-transmission group remains to be interpreted. It may be speculated that access to outpatient units is easier for migrants from outside Europe compared to access to medical practices. Another observation was that patients in hospital-based units showed an inferior clinical CDC stage. A possible explanation is that patients with reduced medical conditions need hospital inpatient care more often and therefore may stay at the hospital’s outpatient unit for further therapy.

The outcome data suggest that disparities between the baseline characteristics of the patient strata in the different medical care facilities do not influence the good overall efficacy of HAART. This may be due to the widespread availability of potent drug combinations, the consideration of baseline resistance testing, the high level of medical expertise of the treating physicians, or good patient adherence. These parameters seem to be more important than medical facility type. Whether these findings can be translated into other countries’ settings is unclear. They show, however, that current antiretroviral treatment in Germany is very successful, independent of other circumstances.

Several limitations of the results should be considered. The large number of cooperating centers and the long period of the study may lead to heterogeneity of methods and applied therapy combinations. This may also be induced by a change of therapeutic concepts and introduction of new antiretroviral compounds during the study period. Furthermore, due to the realization of the study in clinical practice, treatment modifications and patient fluctuations from one medical unit to the other are not included in the analysis. However, considering all study centers were specialized in HIV medicine, the large number of included patients treated in the geographic region of the study [10,12], and the continuously applied study protocol, the limitations may be regarded as limited and the results as valid. For proof of the findings, a prospective randomized trial on the topic is necessary. However, in the authors’ opinion, it is very unlikely that a study with this design would be feasible or find adequate funding. Therefore, the results of this prospective cohort provide the best evidence to date to support the assumption that antiretroviral therapy efficacy is comparable in the two types of medical facilities.

## Conclusion

In summary, the present analysis of the prospective multicenter RESINA study shows that for more than 1,500 patients first-line antiretroviral therapy is very effective for the vast majority of patients independent of medical care setting. This positive finding is even more valid as patient populations differ significantly in baseline parameters relevant for treatment outcome in hospital-based units compared to private practices.

## Appendix

### Cooperating centers in Germany

Peter Arbter, Krefeld; Robert Baumann, Neuss; Ingulf Becker-Boost, Duisburg; Akos-Sigmund Bihari, Wilfried Stücker, Köln; Stefan Esser, Essen; Horst Carls, Frank Huber, Düsseldorf; Stefan Christensen, Münster; Gerd Fätkenheuer, Köln; Beate Gantke, Düsseldorf; Rüdiger Gippert, Peter Hartmann, Hildegard Quaing, Münster; Ingo Greiffendorf, Krefeld; Ute Grüneberg, Münster; Dieter Häussinger, Björn Jensen, Stefan Reuter, Düsseldorf; Petra Hegener, Stefan Mauss, Günther Schmutz, Düsseldorf; Martin Hower, Dortmund; Konrad Isernhagen, Nazifa Qurishi, Katja Römer, Köln; Petra Juretzko, Jürgen Stechel, Köln; Heribert Knechten, Aachen; Wolfgang Köthemann, Anton Neuwirth, Köln; Friedhelm Kwirant, Duisburg; Sabine Mauruschat, Wuppertal; Vladimir Miasnikov, Düsseldorf; Antonius Mutz, Osnabrück; Mark Oette, Tillmann Schumacher, Köln; Michael Paffenholz, Köln; Daniela Petry, Anette Strehlow, Düsseldorf; Michael Radecki, Köln; Martin Reith, Düsseldorf; Jürgen Rockstroh, Bonn; Erhardt Schäfer, Bielefeld; Stefan Schoelzel, Troisdorf; Stefan Scholten, Köln; Theo Scholten, Hagen; Sarah Schons, Düsseldorf; Albert Theisen, Werner Wiesel, Esther Voigt, Köln; and Michael Wichmann, Köln.

## Abbreviations

CDC: Centers for Disease Control and Prevention; CHAIN: Collaborative HIV and Anti-HIV Drug Resistance Network; HAART: Highly active antiretroviral therapy; MSM: Men who have sex with men; NRTI: Nucleoside reverse transcriptase inhibitor; PI: Protease inhibitor; WHO: World Health Organization.

## Competing interests

The authors declare that they have no competing interests.

## Authors’ contributions

MO, SR, RK, TL, and DH contributed to the design of the study. MO, SR, BJ, GF, MH, and AS took part in data collection and analysis. RK, HK, and HP provided resistance testing, results, and interpretation. MO, SR, RK, and DH worked together in manuscript preparation. All authors read and approved the final manuscript.
